# Inside Money, Procyclical Leverage, and Banking Catastrophes

**DOI:** 10.1371/journal.pone.0104219

**Published:** 2014-08-19

**Authors:** Charles D. Brummitt, Rajiv Sethi, Duncan J. Watts

**Affiliations:** 1 Department of Mathematics, University of California Davis, Davis, California, United States of America; 2 Complexity Sciences Center, University of California Davis, Davis, California, United States of America; 3 Department of Economics, Barnard College, Columbia University, New York, New York, United States of America; 4 Santa Fe Institute, Santa Fe, New Mexico, United States of America; 5 Microsoft Research, New York, New York, United States of America; University of Warwick, United Kingdom

## Abstract

We explore a model of the interaction between banks and outside investors in which the ability of banks to issue inside money (short-term liabilities believed to be convertible into currency at par) can generate a collapse in asset prices and widespread bank insolvency. The banks and investors share a common belief about the future value of certain long-term assets, but they have different objective functions; changes to this common belief result in portfolio adjustments and trade. Positive belief shocks induce banks to buy risky assets from investors, and the banks finance those purchases by issuing new short-term liabilities. Negative belief shocks induce banks to sell assets in order to reduce their chance of insolvency to a tolerably low level, and they supply more assets at lower prices, which can result in multiple market-clearing prices. A sufficiently severe negative shock causes the set of equilibrium prices to contract (in a manner given by a cusp catastrophe), causing prices to plummet discontinuously and banks to become insolvent. Successive positive and negative shocks of equal magnitude do not cancel; rather, a banking catastrophe can occur even if beliefs simply return to their initial state. Capital requirements can prevent crises by curtailing the expansion of balance sheets when beliefs become more optimistic, but they can also force larger price declines. Emergency asset price supports can be understood as attempts by a central bank to coordinate expectations on an equilibrium with solvency.

## Introduction

Despite a large and rapidly growing theoretical literature, the mechanisms that give rise to periodic asset price booms and crises remain imperfectly understood. In this paper, we investigate one such mechanism that arises from the interaction between banks and outside investors, in which the capacity of banks to issue inside money—short-term liabilities that are believed to be convertible into currency at par—plays a central role. The model generates a positive correlation between asset prices and bank leverage, which is consistent with the empirical observation that banks, along with other financial intermediaries, tend to finance more of their risky, long-term assets with short-term debt when asset prices rise and less when asset prices fall [1]. That is, bank leverage is procyclical, in contrast with leverage among households and non-financial firms, which is countercyclical. Because a static balance sheet has countercyclical leverage (i.e., a rise in asset prices mechanically lowers leverage, and vice versa), the procyclical leverage of financial intermediaries suggests that they aggressively expand their balance sheets and debt levels when asset prices are rising, and that they contract balance sheets when asset prices are declining.

This rapid expansion and contraction of banks' balance sheets is feasible because the short-term liabilities of banks are viewed by non-banks as close substitutes for currency. The banking sector as a whole can increase its net holdings of risky assets because non-banks are willing to accept these liabilities in exchange for riskier assets [2]. This observation—that the sizes of bank balance sheets can be considerably elastic—is a key element in the model explored here. Another important element is that the objective functions of banks and outside investors differ: we assume that banks operate in a manner that is risk neutral subject to an insolvency constraint, while investors are risk averse. Both groups share a common belief about the future value of certain long-term, risky assets, and they interpret new information about the future value of these assets in the same way. However, because their objectives differ, changes to the common belief about the assets' future value results in trade between the two groups.

For simplicity, we model banks and investors at the sectoral level by considering one consolidated balance sheet for each group. Initially, these two balance sheets are mutually consistent in the sense that neither group wishes to adjust its portfolio at the prevailing prices (given the commonly held belief about the future value of the assets). Whenever this belief changes, trading restores consistency of the balance sheets. In the case of a positive shock to the commonly held belief (representing greater optimism about the future value of the assets), both groups demand more assets at the current prices. Given that the asset supply is fixed in the short term, a positive shock causes prices to rise and portfolios to be adjusted. There exists a unique price at which balance sheet consistency is restored, with assets sold by investors to banks and new short-term liabilities (inside money) created. As a result, banks expand their balance sheets and (typically) increase their leverage.

The response to a negative belief shock is not symmetric. After a negative shock, both banks and investors wish to sell assets at the prevailing prices, but there can be more than one price at which balance sheet consistency is restored. This multiplicity of equilibria results from the bank demand increasing in the asset price: lower prices correspond to greater balance sheet stress and a greater desire to sell. A sufficiently strong negative shock can eliminate two of the equilibria, leaving only one equilibrium, at which the banking sector is insolvent. (In the language of catastrophe theory [3], the set of equilibrium prices undergoes a fold catastrophe as the negative shock becomes more severe. More generally, varying the hypothetical shock using two parameters (rather than one) leads to a cusp catastrophe. A qualitatively similar effect arises in models of stock market crashes induced by dynamic hedging [4].) In short, the unwillingness of outside investors to absorb all the assets that banks wish to sell can cause a discontinuous decline in asset prices and widespread bank insolvency.

We show that successive positive and negative shocks of equal magnitude do not cancel; rather, a banking catastrophe can occur even if beliefs simply return to their initial state. In short, the model is path dependent. A system that can absorb a negative shock of a given size (without suffering a crisis) may be unable to do so if the negative shock is preceded by a positive shock. Optimism induces a buildup of leverage, which increases fragility and can lead to insolvency if the optimism is subsequently reversed.

Finally, within the context of this model we consider the effects of policies such as emergency asset price supports and capital requirements. Support of asset prices by the central bank can coordinate beliefs on an equilibrium that is consistent with bank solvency in the face of asset price declines. Such support was accomplished by the Federal Reserve using a variety of temporary facilities created in 2008; these facilities allowed for the lending of reserves or Treasury securities to financial intermediaries in exchange for much riskier and less liquid collateral. In contrast to emergency policies that deal with an ongoing crisis, capital requirements aim to prevent crises by constraining leverage growth during asset price booms. This constraint can prevent asset price collapses and widespread insolvency when optimism is reversed; however, it can also induce greater selling in a declining market, suggesting the need for graduated or countercyclical constraints on leverage, as has been proposed by the Committee on the Global Financial System [5], Goodhart [6], and Admati and Hellwig [7].

### Related literature

A substantial literature has examined the buildup of fragility in the banking system during periods of low volatility. Long before the most recent crisis, Minsky [8], [9], Crockett [10], and Borio and Lowe [11] argued that periods of stable growth result in changes in financial practices that make subsequent instability more likely. For instance, Crockett ([10] p. 5) argues that in contrast to the conventional wisdom, “it may be more helpful to think of risk as *increasing* during upswings, as financial imbalances build up, and *materializing* in recessions.” Along similar lines, Minsky ([8] p. 126) writes: “Stability—even of an expansion—is destabilizing in that more adventuresome financing of investment pays off to the leaders, and others follow.”

Inside money plays a central role in the theory of credit booms and busts in Dang et al. [12] and Gorton and Ordoñez [13], where emerging doubts about the collateral that backs privately issued short-term debt can cause it to become “information-sensitive”, making investments in determining its quality profitable. Procyclical leverage has been shown to arise from standard risk management strategies based on the concept of Value-at-Risk (VaR), which amount to risk-neutral behavior subject to a constraint on the likelihood of insolvency [14]–[16]. Leverage cycles can also emerge in models of collateral equilibrium [17], [18], in which belief heterogeneity plays a central role; see, for instance, [19] and [20]. In these models, an asset price boom is associated with a distributional shift in wealth towards highly leveraged optimists, with a corresponding reversal when prices collapse. See also Lorenzoni [21] and Bianchi [22], who provide normative analyses of credit booms and busts.

A closely related literature has studied the mechanisms by which a financial crisis spreads. Distress can be transmitted through knock-on contagion, in which the failure of one institution puts its counterparties in a network at risk [23]–[30]. Even without any direct counterparty relationships (e.g., without any loans between banks), the failure of an institution can damage others through the market impact of asset sales—an effect that can be especially strong when institutions' exposures to assets are highly correlated [31]–[33]. Asset correlation, in turn, can arise through the imitation of successful strategies, as in ecological models of financial crises [34]. Contagion can also occur through an information channel, such as when losses in one asset class or financial institution generate worries about related assets and institutions [35]–[37].

Our contribution to this literature is to highlight the asymmetric reaction of balance sheets to positive and negative shocks, the possibility of discontinuous asset price declines, and the path dependence of portfolio adjustments in response to changing beliefs. We make a number of simplifying assumptions that block the mechanisms that have already been extensively explored. First, we assume that the banks and investors are homogeneous, so we model them at the sectoral level as two consolidated balance sheets. Second, we consider only a single class of risky assets, which may be interpreted as securities backed by pools of other assets such as mortgage loans, which in turn are backed by real assets such as housing. Third, we assume that the supply of these assets is fixed, effectively excluding the role of banks in underwriting new assets, a potentially important source of systemic risk. Fourth, we assume that the banks and investors share a common belief about the probability distribution governing the bonds' future value and that they update their beliefs identically in response to new information. Finally, we assume that the short-term liabilities of banks are perceived to be risk-free at all times, so they never become information-sensitive in the sense of Gorton [2]. This assumption rules out the possibility of runs (such as those as in Diamond and Dybvig [38]) and it allows us to focus on asymmetric reactions to shocks, discontinuities in market clearing prices, and the path dependence of portfolio adjustments.

## The Model

### Balance Sheets

Our model proceeds via a sequence of unitless time steps 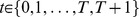. There is a single class of risky assets, henceforth called *bonds*, available in fixed supply. At each step 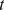, let 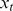 denote the number of bonds held by the banks, where each bond has unit par value (so each bond is worth at most 1 in period 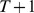). As described in the next section, the market-clearing bond price in period 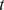, denoted by 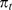, is determined by the commonly-held belief at time 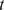 about the bonds' future value. [For simplicity, we assume that no interest payments are made prior to maturity (i.e., these are zero-coupon bonds), so the bond price is bounded above by its par value.] The total value of the banks' bond holdings is therefore 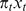. In addition to bonds, the banks' assets also include reserves 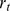, which include vault cash and deposits at the central bank.

Bank liabilities consist of short-term debt that is believed by investors to be convertible into currency at par without risk. We refer to these liabilities as *deposits*, broadly interpreted to include shares in money market funds, which carry an implicit if not explicit government guarantee. These deposits 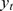 are held by outside investors. The banks' equity at time 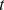 is the residual 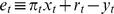. The banks' consolidated balance sheet is shown in Table 1.

**Table 1:** Consolidated bank balance sheet in period *t*.

Bank assets	Bank liabilities
bonds 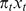	deposits 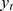
reserves 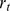	equity 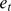

The investors also hold a number of bonds (denoted by 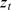), as well as money in the form of currency (denoted by 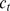) and bank deposits 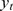. They carry no debt, so their equity 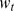 is the sum of the assets 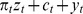. We have in mind real money investors such as pension funds, sovereign wealth funds, insurance companies and retail mutual funds. All leverage therefore exists within the banking system, and we ignore leveraged, non-bank investors such as hedge funds.

For simplicity, we assume that a fixed fraction 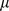 of money in circulation is held by investors as currency, so 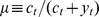 is a parameter of the model, which we restrict to the interval 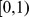. Provided 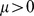, the banks cannot expand their balance sheets without limit because, as described below, they suffer a drain of reserves as they accumulate bonds and because the total quantity of high powered money (currency plus reserves) is in fixed supply, under the control of the central bank. (Our arguments do not rely on 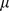 being fixed, only that 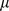 be bounded away from zero at all times, so that the expansion of bank balance sheets results in a drain on reserves.) Table 2 shows the investors' balance sheet.

**Table 2:** Consolidated investor balance sheet in period *t*.

Investor assets	Investor liabilities
bonds 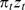	equity 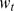
currency 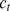	
deposits 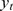	

Throughout this paper, we restrict attention to initial balance sheets that are non-degenerate in the sense that both banks and investors hold some bonds (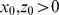) and the banks are solvent (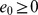).

### Balance sheet consistency

Critical to our analysis is the concept of *balance sheet consistency*, meaning a pair of portfolios and a price of bonds such that banks and investors are both maximizing their respective objective functions, given their shared belief about the bonds' future value. Initially, the banks and investors have balance sheets (i.e., Tables 1 and 2 at time 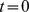) that are consistent with respect to a common belief about the bonds' value. This shared belief changes 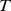 many times via the arrival of unanticipated news that is good or bad. At each time step 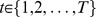, the banks and investors restore consistency of their balance sheets with respect to the new belief at time 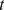 by trading, which results in a new pair of portfolios. This trade also changes the bond price and the money supply, defined as the sum of deposits and currency in circulation (i.e., 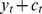). Uncertainty regarding the bonds' value becomes resolved in period 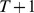.

To define consistency formally, suppose that the banks were to buy 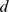 units of bonds from the investors at the price 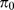. This purchase would require the banks to transfer 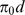 units of money to the investors by giving them 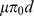 units of currency (drawn from the banks' reserves) and by crediting the investors' deposit accounts by 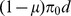 in the aggregate. (An investor who sells bonds to one bank may deposit the proceeds in another bank, but here we model these changes at the consolidated level of the banking sector, ignoring identities of banks.) Table 3 shows the resulting changes to balance sheets.

**Table 3:** Balance sheet changes following a purchase of *d* bonds by the banks at price 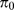 in period *t = *0.

Bank assets	Bank liabilities	Investor assets
bonds 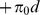	deposits 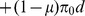	bonds 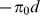
reserves 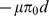		currency 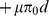
		deposits 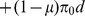

Note that if the banks were to purchase bonds (i.e., if 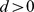), then the money supply (the sum of deposits and currency in circulation) would rise. Conversely, if the banks were to sell bonds (i.e., if 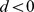), then the money supply would contract.

At each period 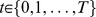, we assume that the banks and investors share a common belief 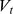 about what each bond will be worth when uncertainty is resolved in period 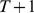. Each belief 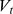 is a continuous random variable with density 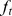 that has support [0, 1] and expected value 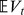.

The banks maximize their expected equity subject to the constraint that their probability of insolvency (when uncertainty is resolved) is smaller than 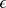, a fixed parameter in (0,1). A popular instantiation of this type of insolvency constraint is known as “Value-at-Risk” or VaR [15]. We note, however, that VaR plays a dual role in the banking system: first, as a tool for risk assessment, where it measures balance sheet strength by estimating the loss in value such that losses greater than it occur with probability no larger than a fixed, small number such as 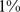; and second, as a strategy for risk management (e.g., setting VaR equal to equity limits the perceived likelihood of insolvency [5]). Here we invoke VaR in this latter sense of risk management.

Meanwhile, the investors maximize their expected utility and are risk averse, with preferences given by a strictly increasing, strictly concave function of terminal wealth 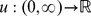.

Now we define balance sheet consistency.

**Definition 1.** *The variables* 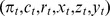 *are* consistent *with respect to the common belief* 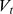 *if and only if*

*1. the banks are maximizing their expected equity subject to their insolvency constraint, i.e.,*

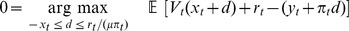





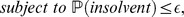

*where* 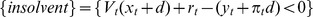; *and*

*2. the investors are maximizing their expected utility*

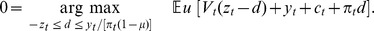



The constraints on *d* in Def. 1, as discussed below, simply ensure that the banks and investors do not sell more bonds than they hold nor buy more bonds than they can finance. The following numerical example, to which we will refer throughout our analysis, illustrates the idea of consistent balance sheets.

**Example 1.** *The balance sheets in* *Table 4* *are consistent with respect to the belief* 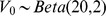 *if* 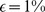 (*i.e., the banks ensure that insolvency occurs with probability at most* 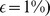 *; the investors hold* 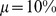 *of their money as currency; and the investors maximize the utility function* 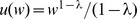 *with* 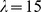 (*i.e., investors' preferences satisfy constant relative risk aversion*). *The bond price is* 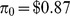 *per unit of face value.*

**Table 4:** Consistent balance sheets for the beliefs and preferences in Example 1.

Bank assets	Bank liabilities	Investor assets	Investor liabilities
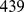 bonds at price 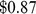	deposits 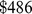	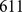 bonds at 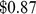	equity 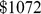
reserves 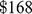	equity 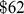	currency 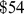	
		deposits 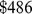	

Next we examine how changes in beliefs affect balance sheets and the market-clearing bond price.

### Belief Shocks

Consider a financial system with a pair of consistent balance sheets at time 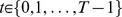, and suppose that new information about the bonds' value emerges in period 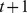 (e.g., news of higher than expected foreclosures in the subprime mortgage market). We assume that this information is public and interpreted identically by the banks and by the investors. Nevertheless, because the banks and investors have different objective functions, the news may cause them to trade and hence reach a new market-clearing price. We consider shocks to the belief 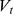 that shift probabilities to higher values (positive shocks) or to lower values (negative shocks).

**Definition 2.** 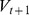 *is a* positive shock *to* 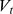 *(or equivalently* 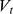 *is a* negative shock *to* 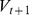 *) if and only if*

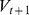
 *second-order stochastically dominates* 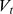 *[i.e.,* 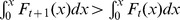 *for all* 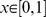 *, where* 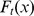 *is the CDF of* 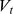 *], and*

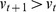
 *, where* 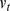 *is the first* 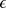 *-quantile of* 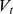 *[i.e., the inverse of the CDF of* 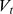 *evaluated at* 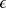 *, i.e.,* 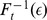 *].*

To determine the changes in the portfolios and the change in the bond price that would restore consistency after a shock, we express the investors' demand for bonds 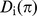 and the banks' demand for bonds 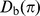 in terms of a hypothetical price 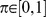, given the consistent balance sheets in period *t*. A market-clearing price must make excess demand vanish in the aggregate, so the new market-clearing price 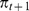 is a root of the total demand 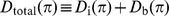. The banks' and investors' demands at this price determine their new portfolios.

### Investor Demand

As in the definition of consistency (Definition 1), after the belief 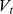 is replaced by 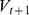, the investors adjust portfolios to maximize the expected value of their utility. Because the proportion of the investors' money in currency before and after the purchase of bonds is 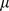, the investors can buy 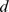 units of bonds using 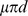 dollars in cash and 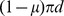 dollars from their deposit accounts. Investors cannot borrow, so their total demand is upper-bounded by their holdings of money (currency plus deposits). That is, investors can demand at most 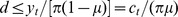 many bonds. Also, the investors cannot sell more bonds than they hold, so they must demand at least 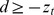. Thus the investors demand
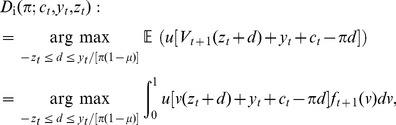
(1)where 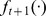 is the probability density of 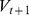. Lemma S1 in File S1 shows that, under mild assumptions satisfied here, the investors' demand (1) is single-valued at all prices (and hence can be expressed as a function).

Following Example 1, we consider the special case with investor utility 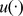 that satisfies constant relative risk aversion (CRRA) and Beta-distributed beliefs. That is, the investors' utility function 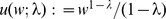, where 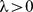 and 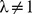, and the beliefs of bond values at time 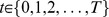 are 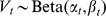 for some 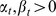.

**Figure 1:**
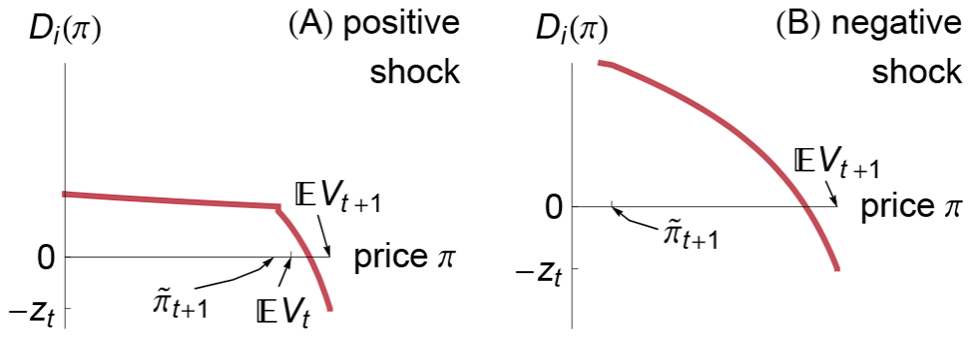
Illustrations of the investors' demand function for positive and negative shocks. Immediately after a positive shock [panel (A)], the investors wish to buy bonds at the current price, but they are willing to sell bonds if the price increases enough. Immediately after a negative shock [panel (B)], the investors wish to sell bonds at the current price, but they are willing to buy bonds if the price decreases enough. In general, the investor demand function 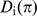 [defined in Eq. (1)] is non-increasing in the price 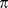. By Lemma S1 in File S1, we know that the investors demand 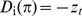 if and only if 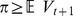. Here, investor preferences are represented by the CRRA utility function 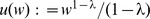 for 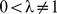; for this case, we know by Lemma S2 in File S1 that the investors buy as much as they can afford, 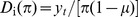, if and only if 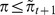.

Figure 1 illustrates that in this case the investor demand function 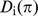 is non-increasing with the price 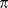, and there is some price strictly below 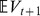 above which demand becomes negative. Lemma S2 in File S1 shows that the investors' demand falls below 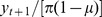, the maximum number of bonds that the investors can afford, precisely at the price 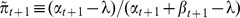.

### Bank Demand

Recall that the banks maximize their expected equity subject to an insolvency constraint. Consider a hypothetical price 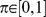 after the shock that replaces the belief 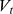 by 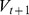. If this hypothetical price 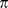 would render the banks insolvent (i.e., if 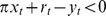), then the banks are forced to sell all their bonds, so they demand 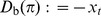. Otherwise, the banks remain solvent, so they demand a number of bonds 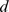 that maximizes their expected equity in period 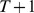 using the new belief 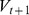,
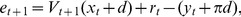
(2)subject to the three constraints



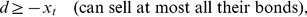
(3a)

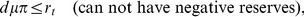
(3b)




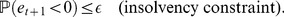
(3c)


Thus, the banks' demand function is
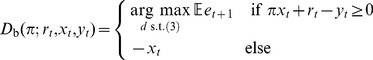
(4)


Next we simplify the insolvency constraint (3c). Define 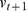 as the first 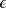-quantile of the belief 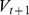. That is, under belief 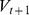, the probability that the terminal value of bonds will be at least 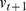 is 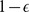. Because 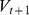 is continuous, the insolvency constraint (3c) is equivalent to
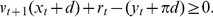
(5)


From (5), we see that the banks poise themselves at 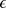-probability of insolvency [i.e., 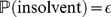] by demanding
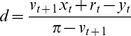
(6)many bonds, provided that 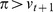.

In Appendix S2 in File S1, we combine Eq. (6) with the constraints (3a) and (3b); here we give the result. If 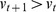, as occurs in a positive shock, then the numerator 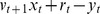 of Eq. (6) is positive, so the banks satisfy their insolvency constraint (5) after the shock. Then the banks want to buy bonds according to the decreasing function
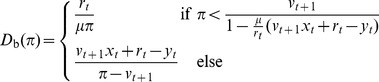
(7a)as long as the banks are currently solvent (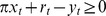) and the price 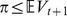.

**Figure 2:**
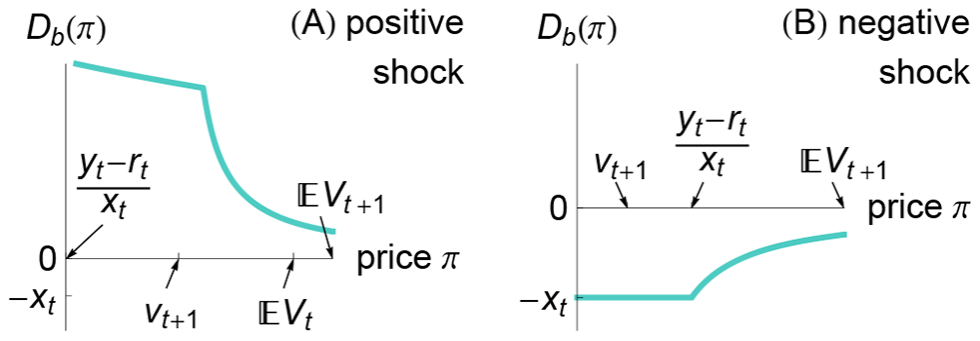
Illustrations of the banks' demand function for positive and negative shocks. Immediately after a positive shock [panel (A)], the banks comply with their insolvency constraint (5) (i.e., 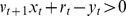); in this case, the banks want to buy bonds provided that the price 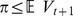 and provided that the banks are currently solvent (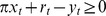); the banks' demand is given by Eq. (7a). Immediately after a negative shock [panel (B)], the banks violate their insolvency constraint (5) (i.e., 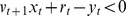); in this case, the banks want to sell bonds, and the banks' demand is given by Eq. (7b). The plot ranges are identical in Figs. 1(A) and 2(A) and in Figs. 1(B) and 2(B); the sums of these demands are shown in Fig. 3.

On the other hand, if 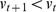, as occurs in a negative shock, then the numerator 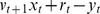 of Eq. (6) is negative, meaning that the banks violate their insolvency constraint (5) immediately after the shock. Then the banks will sell bonds at all prices by demanding
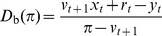
(7b)as long as the banks are currently solvent (i.e., 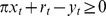) and the price 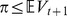. Notice in Eq. (7) and in Fig. 2(B) that for negative shocks the banks' demand *increases* with the price 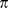. In contrast to typical demand functions, the banks sell *more aggressively* at *lower* prices. This increasing demand function results in a non-monotonicity of aggregate demand in the face of negative shocks.

### Aggregate Demand

**Figure 3:**
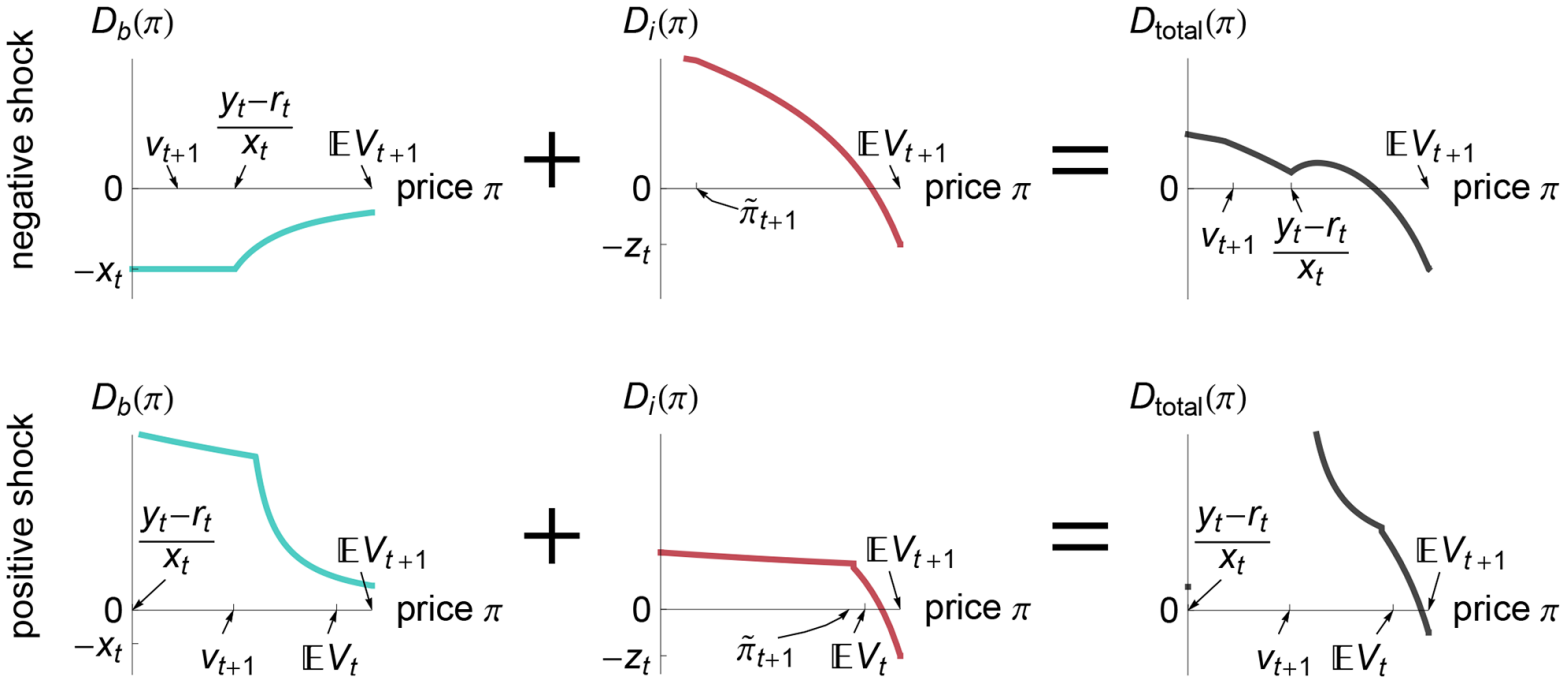
Illustrations of the total demand for a negative shock (top row) and for a positive shock (bottom row). The total demand is the sum of the bank demand function 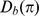 (left column) and the investor demand function 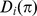 (middle column). Within each row, the plot ranges are identical. The plots in the left and middle columns are identical to Figs. 1–2.

In Fig. 3, we plot the total demand function 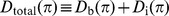, the roots of which are the new equilibrium prices. The plot ranges in the top and bottom rows of Fig. 3 are identical. Notice that the banks' demand in the case of a negative shock (top-left plot of Fig. 3) is increasing in the bond price, which can make the total demand (top-right plot of Fig. 3) non-monotonic and hence have multiple roots (i.e., multiple equilibrium prices 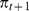). Next we show that at one of those multiple equilibria the banks are insolvent and that banking crises can appear “out of the blue”.

## Results

### Negative Shocks

**Figure 4:**
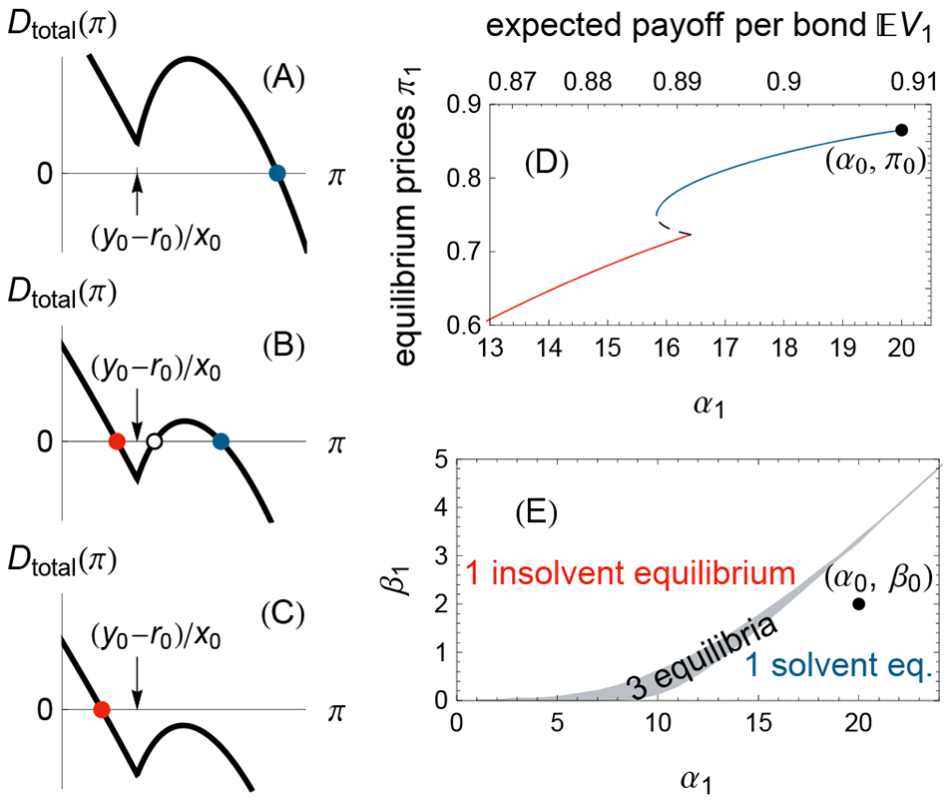
Cusp catastrophe from a negative shock. The initial condition is Example 1, in which the initial belief 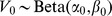 with 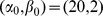. In panels (A)–(D), we consider negative shocks 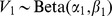 for progressively smaller 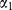, with 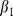 fixed at 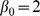. Panels (A)–(C) show the total demand function 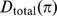, the roots of which are the market-clearing prices 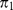; filled and empty circles in (A)–(C) denote stable and unstable equilibria, respectively; the banks are solvent at an equilibrium price 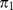 if and only if 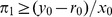. The following behaviors occur as 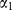 decreases: the negative shock becomes more severe because the expected payoff from bonds decreases according to 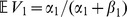 [see the top axis of (D)]; the largest equilibrium price [the right-most root 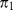 in (A)–(C), the maximum of the curves in (D)] decreases, at first slowly and then precipitously when 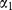 passes the region of three equilibria [compare panels (B) and (C) and notice the region of three equilibria near 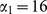 in (D)]; after passing the region of three equilibria, there exists only one equilibrium, at which the banks are insolvent [red dot in (C), red curve in (D)]. The gray region in (E) marks the belief 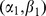 that gives rise to three equilibria [as in (B)]; above that region, the price drops sharply, and the banks are insolvent, i.e., 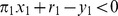 [see (C)].

Starting from a pair of consistent balance sheets, a small negative shock to beliefs would lead to a slightly smaller bond price. We illustrate that market-clearing price using a blue dot in Fig. 4(A). A slightly stronger negative shock could create two new equilibrium prices, marked by a red dot and by a black circle in Fig. 4(B). Those two new prices appear in a saddle-node bifurcation at the price 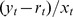. An even stronger negative shock annihilates the two larger equilibrium prices in another saddle-node bifurcation [Fig. 4(C)]. Stable and unstable roots of 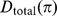 are depicted by filled and empty circles, respectively, in Fig. 4.

The price 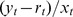 marks not only where two new equilibrium prices appear but also whether the banks become insolvent, because the banks are solvent at a hypothetical price 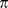 if and only if 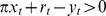. The prices marked by red dots in Figs. 4(B)–(C) and by the red line in Fig. 4(D) are equilibrium prices that result in an insolvent banking sector [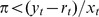], whereas the blue dots in Figs. 4(A)–(C) and the blue line in Fig. 4(D) denote prices that maintain solvency.

Figures 4(A) and 4(B) demonstrate that a small shock can create an equilibrium at which the banking sector is insolvent [i.e., create the red dot in Fig. 4(B)] even if there continues to exist an equilibrium price close to the original price and at which the banks are solvent [i.e., the blue dot exists in Fig. 4(B)]. This multiplicity of equilibria implies that a widespread fear of a crisis can become self-fulfilling and result in a collapse of asset prices, despite the fact that no crisis would occur in the absence of such fear. Actions by the central bank in supporting asset prices and in containing fear can effectively coordinate expectations on the more optimistic equilibrium [the blue dot in Fig. 4(B)]. However, if the shock is sufficiently large, as in Fig. 4(C), then coordinating expectations alone cannot prevent crisis because there no longer exists an equilibrium with a solvent banking sector.

In summary, negative shocks lead to a cusp catastrophe [3]. Specifically, there is a region of shocked beliefs 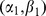, marked in gray in Fig. 4(E), that give rise to three equilibrium prices. Uniqueness of equilibrium is restored for beliefs above that gray region, but this equilibrium is a very different kind because it entails widespread bank insolvency. Next, we show that positive shocks increase the risk of such a crisis.

### Positive Shocks

Now suppose that beliefs about the terminal value of the bonds becomes more optimistic because, for example, the prices of underlying assets (e.g., housing prices) rise faster than expected. This positive shock generates slack in the insolvency constraint (3c), which allows the banks to undertake more risk by buying bonds and by expanding their balance sheets. Because the investors share the more optimistic beliefs, they also wish to buy bonds at the price that prevailed before the arrival of the positive shock. Thus, there is excess demand for bonds at the initial price, so the price will accordingly rise. (Recall, our model assumes that the total volume of bonds is fixed, thus ruling out the possibility that banks can increase the size of their balance sheets by underwriting new assets.)

The price will rise until the investors are enticed to sell bonds to the banks. To see why, recall that after a positive shock the banks demand to buy bonds at all prices below the new expected value 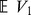 [recall Fig. 2(A)]. The investors, meanwhile, demand to sell bonds if and only if the price becomes large enough [recall from Fig. 2(A) that their demand becomes negative just below 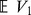]. Thus, the new market-clearing price will be determined by the investors selling bonds to the banks. The banks correspondingly expand their balance sheets and increase the money supply as they credit the investors' deposit accounts.

**Figure 5:**
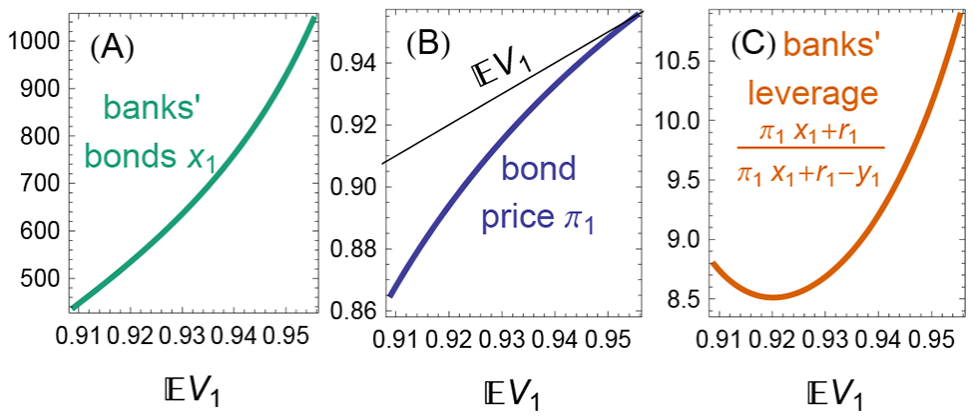
Effect of a positive belief shock. A positive belief shock induces the banks to buy bonds (A), to bid up the bond price (B), and typically to increase their leverage (C). Here, we subject Example 1 to a belief shock 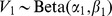, for 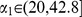 and 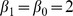. The axis 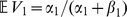 is the severity of the positive shock.

As the positive shock becomes stronger (larger expected bond value 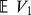), the banks buy more bonds [Fig. 5(A)] at an ever higher price 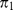 [Fig. 5(B)]. Bank leverage (the ratio of assets to equity) rises if the news is sufficiently positive [Fig. 5(C)]. Next, we show that this procyclical leverage worsens the risk of bankruptcy when beliefs subsequently become pessimistic.

### Reversal of a Positive Shock

Consider a positive shock followed by a negative shock that restores the initial belief. One might expect that such shocks of equal magnitude “cancel” and leave the financial system unchanged. However, this model is nonlinear, so portfolios depend on the history of beliefs. Moreover, a sufficiently strong positive shock [that results in greater bank leverage; see Fig. 5(C)] that is followed by an equally strong negative shock can result in insolvency.

**Figure 6:**
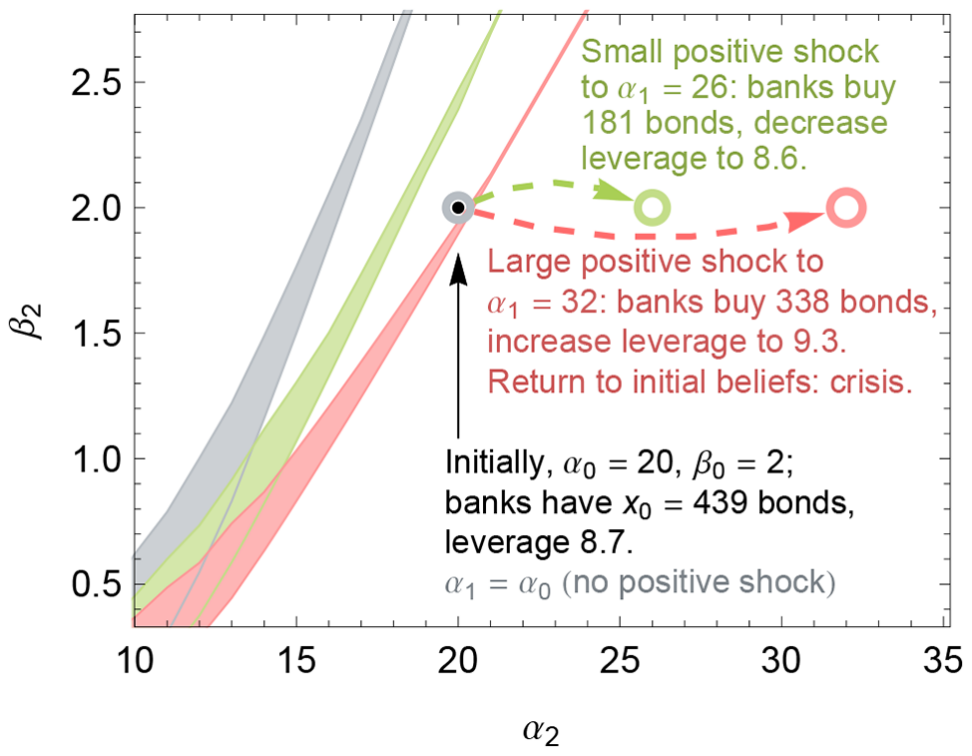
Crisis from reversing a positive shock. A sufficiently strong positive belief shock followed by an equally negative shock can result in insolvency. Here, the initial condition is given by Example 1, in which the initial belief 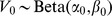 with 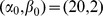, marked by a black dot. At time 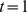, a positive shock changes the belief to have parameters 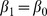 and 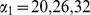 (gray, green, and red circles, respectively). The horizontal and vertical axes are the parameters 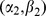 of the new belief at time 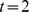. The three colored regions denote the belief parameters 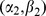 such that there exist three equilibrium prices 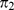. The gray region is identical to that in Fig. 4(E). As in Fig. 4(E), above the colored region, the banks are insolvent at the unique equilibrium price. Note that the black dot lies above the red region, meaning that restoring the initial beliefs after a large positive shock to 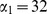 (red circle) leads to insolvency.

Figure 6 illustrates such a crisis caused by optimism that is reversed. The initial belief 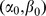 is marked by a black dot. At step 1, the belief undergoes either a small positive shock, a large positive shock, or no shock at all; the resulting belief parameters 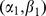 are marked in Fig. 6 by the green circle, red circle, and gray circle, respectively. Next, consider in step 2 a second shock to a belief 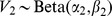. The colored regions in Fig. 6 are the regions of beliefs 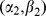 that give rise to three equilibrium prices [just like Fig. 4(E)].

We focus in particular on what happens if the initial belief is restored in period 2, i.e., if 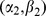 equals 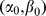, the black dot. In this case, the initial and final beliefs are identical; we only vary the intermediate belief in period 1. This intermediate state crucially moves the region of multiple equilibria for period 2. For the small positive shock (the green circle and green region in Fig. 6), the initial belief (black dot) lies to the right of the green region; thus, restoring the initial belief after the small positive shock does not threaten the banks' solvency. However, for the large positive shock (the red circle and red region), the initial belief lies to the *left* of the red region, so restoring the initial belief after the larger positive shock moves the financial system across the region of multiple equilibria and into the region of certain insolvency. To reiterate, reversing a large positive shock can precipitate widespread bank insolvency. This behavior is consistent with a number of historical episodes, including the global financial crisis of 2007–2009, in which a rise and subsequent fall in real estate prices led to the near collapse of the banking system.

Up to this point we have assumed that banks are unconstrained by regulatory requirements. Next we consider the effects of capital requirements.

### Capital Requirements

A capital requirement aims to prevent insolvency crises by requiring that banks maintain sufficient levels of capital (equity) relative to their risk-weighted assets. In accordance with the standards in Basel III, we assign risk weight zero to bank reserves and risk weight one to the banks' bond holdings. With this assumption, the banks' *capital-to-assets ratio* 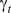 is the banks' equity 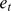 divided by the total, market-value of their bond holdings, 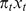. Then the *capital requirement* mandates that this capital-to-assets ratio 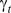 equals or exceeds some minimum amount 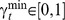,
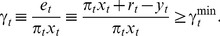
(8)at each step 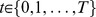


Next we examine the effect of this constraint on the banks' demand in the events of positive and negative shocks.

#### Capital Requirements and Bank Demand

If the banks were to buy *d* bonds at a new price 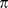 in period 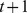, then the balance sheets would become those in Table 3, as discussed above. The capital requirement (8) gives another constraint on the banks' demand *d*,
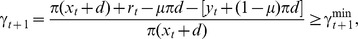
or, upon rearranging,



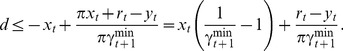
(9)In the event that this upper bound (9) on the banks' demand 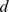 is smaller than 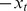, we define the capital constraint to be 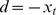 because the banks cannot sell more bonds than they hold. Thus, the cap on bank demand due to the capital requirement, denoted by 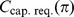, is defined to be
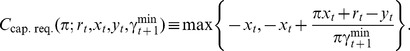
(10)


Augmenting the banks' demand function (4) with the capital constraint 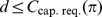 results in the demand function
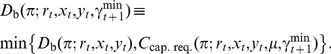
(11)


If 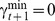, then the bank demand (11) reduces to the original one in Eq. (4).

Next we show that capital requirements can act as a stabilizing force in the event of positive shocks, but can also be destabilizing when negative shocks occur.

#### Stabilizing Effects of Capital Requirements

**Figure 7:**
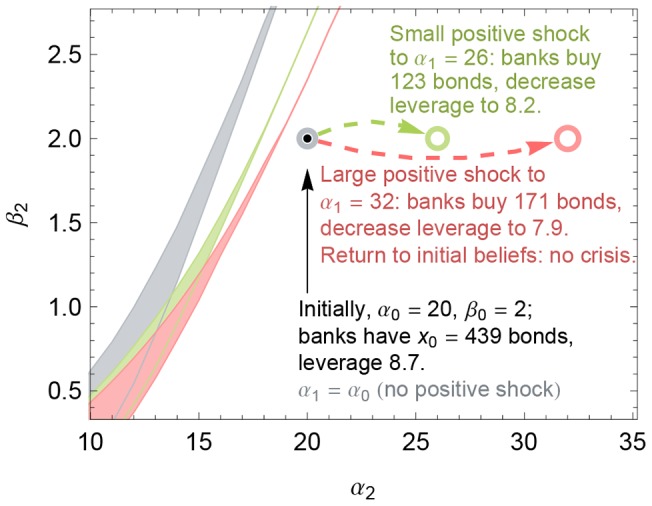
A capital requirement can prevent insolvency when a positive shock is followed by a negative shock. Here, we repeat the experiments in Fig. 6 with a capital requirement of 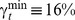 (rather than 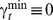). The banks buy fewer bonds after the positive shocks to 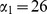 (green circle) and to 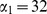 (red circle). Consequently, when a second shock occurs, the regions of beliefs 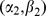 that give rise to three equilibria (i.e., the green and red regions) move to the right less than they do when there is no capital requirement, so the system is less vulnerable to insolvency if a negative shock subsequently occurs in period 2. Unlike in Fig. 6, the black dot lies *below* the red region, meaning that restoring the initial beliefs in period 2 after the large positive shock in period 1 does not lead to insolvency.

**Figure 8:**
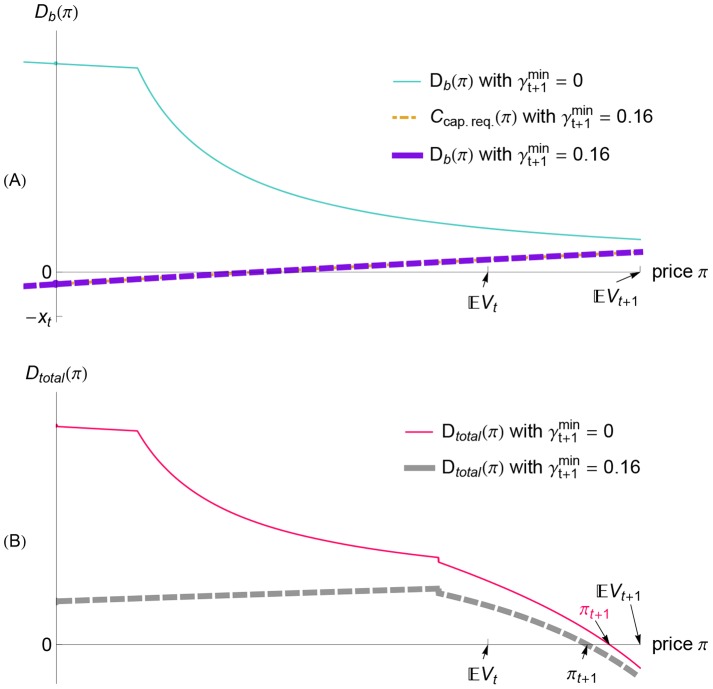
When a positive belief shock occurs, a capital requirement can limit the banks' bond purchases. Here, we show the bank demand [panel (A)] and the total demand [panel (B)] for the case of a positive shock to Example 1 from 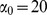 to 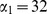 with a capital requirement of 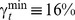 (i.e., the positive shock in the red case in Fig. 7). The capital requirement binds for all prices shown in panel (A), so the bank demand 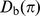 (purple dashed line) equals the capital constraint 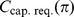 (orange dot-dashed line). Because the banks' demand is reduced in order to satisfy the capital requirement [panel (A)], the total demand is reduced, as shown by the gray dashed line in panel (B). Consequently, the new equilibrium price in the case of a capital requirement of 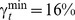 (labeled 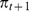 in black) is smaller than the new equilibrium price without a capital requirement (labeled 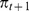 in pink). Thus, due to the capital requirement, the banks buy fewer bonds, and the bond price increases less, so the system is less vulnerable to insolvency if negative shocks subsequently occur (Fig. 7).

As noted above, a capital requirement effectively places a limit on banks' bond purchases when a positive shock occurs. An obvious consequence is that it also limits the corresponding growth in banks' leverage and hence their vulnerability to insolvency in the event of a subsequent negative shock. To illustrate this behavior, we repeat the experiments in Fig. 6 (i.e., a positive shock to Example 1 followed by a negative shock), but this time we implement a capital requirement with 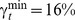 at every step *t*. (This capital requirement nearly binds in step 0 because the initial capital-to-assets ratio in Example 1 is 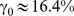, just barely above the minimum of 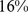.) Figure 7 shows the result. First, the banks purchase fewer bonds in the positive shocks because of the capital requirement. (Figure 8 shows how the capital requirement affects the demand functions for the severe positive shock in Fig. 7.) Because the banks buy fewer bonds in the positive shock, the region of three equilibria does not move as much to the right as they did without a capital requirement (compare the green and red regions in Fig. 7 with those in Fig. 6), so the banks are less vulnerable to insolvency in the event of a subsequent negative shock. In fact, the red region now lies *above* the initial belief parameters 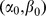, which is marked by a black dot in Fig. 7.

Recall that, without a capital requirement, this large positive shock led to an insolvency crisis if it was reverted (Fig. 6); by contrast, with a capital requirement of 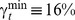, reverting this large positive shock does not lead to an insolvency crisis. In other words, our result is supportive of recent arguments for countercyclical capital requirements [5], which maintain that capital requirements be increased during boom periods and relaxed during crises—a point that is further elaborated in the following section.

#### Destabilizing Effects of Inflexible Capital Requirements

Although capital requirements can help banks avoid insolvency in the event of positive-negative shock combinations, inflexible capital requirements can also be problematic. In particular, for some negative shocks the capital requirement specified in Eqn. (10) can eliminate the two large-price equilibria (at which the banks would have remained solvent) by forcing the banks to demand to sell even more bonds at those prices. With the two large-price equilibria eliminated, only a small-price equilibrium remains, and the banks are insolvent at this equilibrium. In other words, the capital requirement can force the bond price to collapse and the banks to become insolvent.

**Figure 9:**
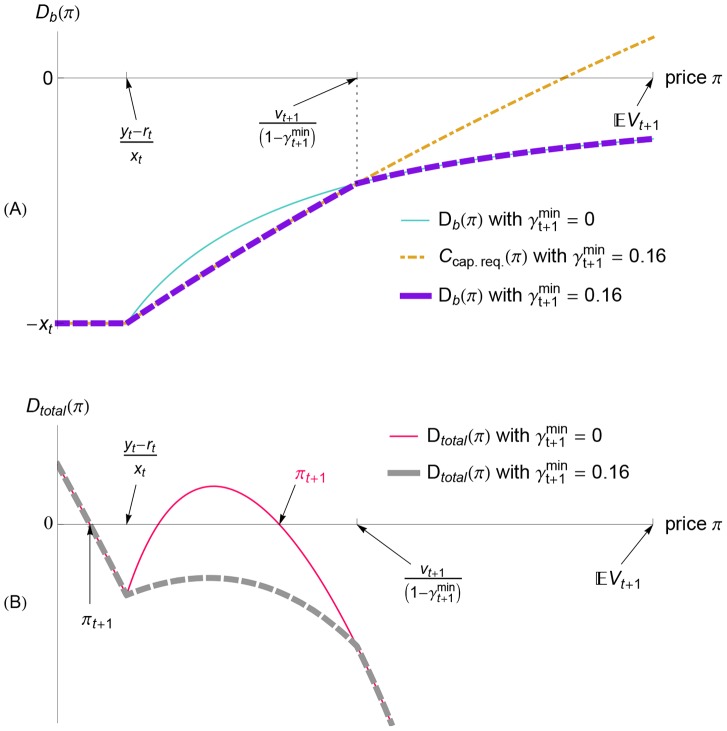
When a negative belief shock occurs, a capital requirement can force the banks to sell bonds more aggressively. This pressure can be so strong that it causes a large price decline. Here, we illustrate the effect of a capital requirement on the banks' demand function 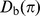 [panel (A)] and on the total demand function 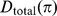 [panel (B)]. The thin lines in panels (A), (B) are the demand functions for the case of no capital requirement (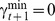), while the thick, dashed lines are the demand functions for a capital requirement of 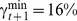. Because of the capital requirement (8), the banks' demand cannot exceed 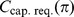 [given by Eq. (10) and plotted as an orange dot-dashed line in panel (A)]. Consequently, the banks are forced to sell more bonds in order to satisfy the capital requirement if the price 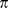 is sufficiently small. Thus, in panel (B) we see that implementing the capital requirement reduces the total demand 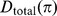 over a certain range of prices 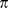. In some cases, as in the case shown in panel (B), the capital requirement can cause the equilibrium price to decline dramatically: compare the market-clearing price when there is no capital requirement (the horizontal tick labeled 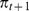 in pink) with the market-clearing price when there is a capital requirement of 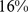 (the horizontal tick labeled 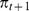 in black). Further note that this dramatic decline in the bond price causes the banking sector to be insolvent, because 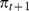 (for 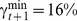, marked in black) is smaller than 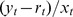. The numerical values in this example are a negative shock from 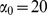 to 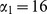, with initial condition given by Example 1 (for which 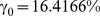).

Figure 9 shows an example of such a negative shock that becomes a crisis if there is a capital requirement. Without a capital requirement, there are three equilibrium prices because the total demand function 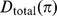 [the pink, thin line in the plot of the bank demand in Fig. 9(B)] has three roots. Implementing a capital requirement truncates the banks' demand over the prices 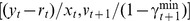, as shown in Fig. 9(A). Consequently, with a capital requirement, the two large-price equilibria have disappeared, leaving only a small equilibrium price that entails bank insolvency [see the unique root of 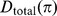 with no capital requirement, given by the thick, dashed curve in Fig. 9(B)].

**Figure 10:**
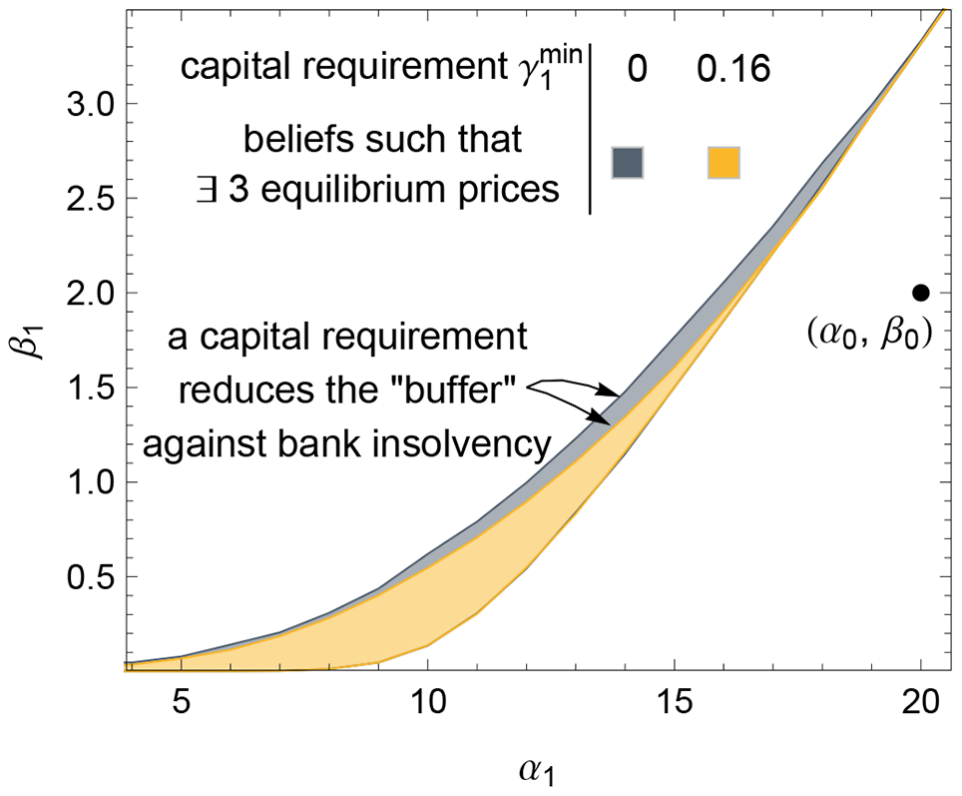
Capital requirements can cause bank insolvency in the event of a negative shock. The region of shocked beliefs giving rise to three equilibrium prices (marked by a color region) shrinks from above when one implements a capital requirement. [Here, the initial condition is Example 1, for which initial capital adequacy ratio 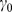 is 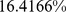, and we implement a capital requirement of 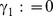 (gray region) or 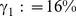 (yellow region).] This region of three equilibria acts as a buffer against bank insolvency, so the capital requirement reduces this buffer. To see this conclusion, consider a pair of belief parameters 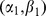 that lie in the gray region but not in the yellow region; if there is a capital requirement, then the bond price declines dramatically in period 1 and the banks become insolvent, but if there were no capital requirement then no such crisis would occur. [Figure 9 shows the demand functions for one such example with 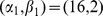.]

This event (that a capital requirement causes an insolvency crisis) is rather generic: Fig. 10 shows that implementing a capital requirement shrinks the region of beliefs giving rise to three equilibrium prices. More specifically, a less severe negative shock suffices to move the financial system above the region of three equilibria and into the region of certain bank insolvency. The region of three equilibria acts as a “buffer” against bank insolvency, and the capital requirement shrinks that buffer. [For details on why it shrinks the region of three equilibria *from above* (and not from below), as illustrated in Fig. 10, see Appendix S3 in File S1.]

These destabilizing effects substantiate recent arguments for capital requirements that are graduated or flexible, as argued by Admati and Hellwig ([7] p.189) and by Goodhart [6]. Basel III has taken a step in this direction by implementing two requirements: if a bank's equity lies between 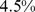 and 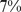 of its risk-weighted assets, then the bank is required to slowly rebuild equity by retaining profits and by avoiding paying dividends, but the bank need not raise new equity immediately [7].

## Conclusion

In focusing on inside money and procyclical leverage, our model excludes several other potentially important sources of systemic risk. For example, our assumption that the banks and investors are homogeneous, and hence described by two consolidated balance sheets, effectively rules out knock-on contagion. Asset-to-asset contagion cannot occur because we consider only a single risky asset. Market failure due to information asymmetry cannot occur because of our assumption that the banks and investors share a common belief about the asset's value, and they update their beliefs identically in response to new information. Finally, bank runs cannot occur because we assume that the bank liabilities are implicitly or explicitly guaranteed.

Although these simplifying assumptions make our model unrealistic in many respects, they also highlight how little complexity is needed to generate systemic risk; specifically, the combination of inside money and procyclical leverage suffices to generate crises. Moreover, the model provides a framework for understanding the effects of policies such as leverage constraints and emergency asset price supports. Many of the historically unprecedented actions taken by the Federal Reserve in 2008 involved attempts to support asset prices that the central bank could not directly purchase. By creating facilities that accepted these assets as collateral in exchange for loans of reserves or Treasury securities, the Fed may have been trying to coordinate beliefs on more optimistic equilibria with a solvent banking sector. This action was a response to crisis conditions that arose in part because of excessive leverage, which countercyclical capital requirements could have held in check. Our model demonstrates the need for both types of policy in order to mitigate or prevent the catastrophic consequences of a reversal of optimism.

## Supporting Information

File S1 **Supporting files. Appendix S1, Properties of the investor demand function. Assumption S1. Assumption S2. Assumption S3. Assumption S4. Lemma S1. Lemma S2. Appendix S2, Derivation of the bank demand function. Appendix S3, Effect of a capital requirement on the region of beliefs giving rise to three equilibria.**
(PDF)
