# Supplementary material for: Inside Money, Procyclical Leverage, and Banking Catastrophes
Source: PLoS One. 2014 Aug 19;9(8):e104219. doi: 10.1371/journal.pone.0104219 (PMC4138099; doi:10.1371/journal.pone.0104219)
Supplement: File S1 — Supporting files. Appendix S1, Properties of the investor demand function. Assumption S1. Assumption S2. Assumption S3. Assumption S4. Lemma S1. Lemma S2. Appendix S2, Derivation of the bank demand function. Appendix S3, Effect of a capital requirement on the region of beliefs giving rise to three equilibria. (PDF) [file pone.0104219.s001.pdf]

Inside Money, Procyclical Leverage, and Banking Catastrophes: Supporting Information (SI)

Charles D. Brummitt[1,2,*], Rajiv Sethi[3,4], Duncan J. Watts[5]

1 Department of Mathematics, University of California, Davis, CA 95616 USA

2 Complexity Sciences Center, University of California, Davis, CA 95616 USA

3 Department of Economics, Barnard College, Columbia University, New York, NY 10027

4 Santa Fe Institute, Santa Fe, NM 87501 USA

5 Microsoft Research, New York, NY 10011 USA

∗ E-mail: Corresponding author cbrummitt@math.ucdavis.edu

Appendix S1 Properties of the investor demand function

Here, we formalize and prove properties of the investors' demand function that were mentioned in the text. Lemma S1 shows that the investor demand is single-valued, under the following mild assumptions on the investors' utility function and on the random variable representing beliefs about the bonds' value. Next, Lemma S2 achieves stronger results for the particular utility function with constant relative risk aversion (CRRA).

Lemma S1–S2 make the following assumptions. (All four assumptions except for Assumption S2 and the second part of Assumption S3 were mentioned in the main text.)

Assumption S1. *The investors' utility function* $u : (0, \infty) \to \mathbb{R}$ *is strictly increasing and strictly concave.*

Assumption S2. *The investors' utility function* $u(\cdot)$ *is twice continuously differentiable.*

Assumption S3. V_{t+1} *is an absolutely continuous random variable, and its density function* f_{t+1} *is a continuous function on* $[0, 1]$.

Assumption S4. *The investors have some bonds* ($z_t > 0$)*, some deposits* ($y_t > 0$)*, and some currency* ($c_t > 0$).

Some elementary results in convex optimization imply that the investors' demand is single-valued and that they want to sell all their bonds if and only if the price equals or exceeds the expected value of bonds.

Lemma S1 (Investor demand is single-valued and equals $-z_t$ iff price $\pi \geq \mathbb{E}\, V_{t+1}$)**.** *Under Assumptions S1–S4, the investor demand function is single-valued. Furthermore,* $D_i(\pi) = -z_t$ *if and only if* $\pi \geq \mathbb{E}\, V_{t+1}$.

Proof. Let $z_t, y_t, c_t > 0$ (by Assumption S4), and let the currency fraction $\mu \in [0, 1)$ and the hypothetical price $\pi \in (0, 1)$. Recall that the investors' demand, Eq. (1) of the main text, is defined to be

$$D_i(\pi; c_t, y_t, z_t) := \underset{-z_t \,\leq\, d \,\leq\, y_t/[\pi(1-\mu)]}{\arg\max} \; \mathbb{E}\, \left(u\left[V_{t+1}(z_t + d) + y_t + c_t - \pi d\right]\right). \tag{S1}$$

For convenience, let $g(d; z_t, y_t, c_t, \pi)$ denote the objective function of $D_i(\pi)$, i.e.,

$$\begin{aligned} g(d; z_t, y_t, c_t, \pi) &:= \mathbb{E}\, \left(u\left[V_{t+1}(z_t + d) + y_t + c_t - \pi d\right]\right) \\ &= \int_0^1 u\left[v(z_t + d) + y_t + c_t - \pi d\right] f_{t+1}(v) dv, \end{aligned}$$

The domain of g is the interval $[-z_t, y_t/(\pi(1-\mu))]$, which is convex.

The continuity of u and u' (by Assumption S2) and of f_{t+1} (by Assumption S3) enable us to use the Leibniz integration rule twice to move the derivative inside the integral sign to compute the first two derivatives

$$\frac{\partial g}{\partial d} = \int_0^1 u'\left[v(z_t + d) + y_t + c_t - \pi d\right](v - \pi) f_{t+1}(v) dv, \tag{S2a}$$

$$\frac{\partial^2 g}{\partial d^2} = \int_0^1 u''\left[v(z_t + d) + y_t + c_t - \pi d\right](v - \pi)^2 f_{t+1}(v) dv. \tag{S2b}$$

Because u is strictly concave (by Assumption S1) and twice differentiable (by Assumption S2), and because its domain $(0, \infty)$ is convex, we know that $u'' < 0$ [1, Sec. 3.1.4, page 71]. Also, we know that $(v - \pi)^2 \geq 0$ with equality if and only if $v = \pi$. Combining these two conclusions with Eq. (S2b) gives

$$\frac{\partial^2 g}{\partial d^2} < 0 \quad \text{for all } d \in \left[-z_t, \frac{y_t}{\pi(1-\mu)}\right]. \tag{S3}$$

Because $\partial^2 g/\partial d^2 < 0$ and because the domain of g is convex, we know that $g(d)$ is strictly concave [1, Sec. 3.1.3, page 69]. Thus, any solution d^* to the first-order (necessary) condition for the maximization in

Eq. (S1),

$$\frac{\partial g}{\partial d}(d^*; z_t, y_t, c_t, \pi) = 0, \tag{S4}$$

is a unique, global maximum [1, Sec. 3.1.3, page 69]. If no solution d^* to Eq. (S4) exists, then $\partial g/\partial d$ is either positive for all d, in which case $g(d)$ has a unique maximum at $-z_t$, or $\partial g/\partial d$ is negative for all d, in which case $g(d)$ has a unique maximum at $y_t/[\pi(1-\mu)]$. Thus, the investor demand function (S1) is single-valued.

Furthermore, the first derivative of the objective function evaluated at the lower constraint $d = -z_t$ is

$$\frac{\partial g}{\partial d}(-z_t; z_t, y_t, c_t, \pi) = (\mathbb{E}\, V_{t+1} - \pi)u'(y_t + c_t + \pi z_t).$$

Because $u' > 0$ (by Assumptions S1–S2), we know that

$$\frac{\partial g}{\partial d}(-z_t; z_t, y_t, c_t, \pi) \le 0 \quad \text{if and only if} \quad \pi \ge \mathbb{E}\, V_{t+1} \tag{S5}$$

Equations (S5) and (S3) imply that $D_i(\pi) = -z_t$ if and only if $\pi \ge \mathbb{E}\, V_{t+1}$, which completes the proof. □

The next lemma achieves stronger results for the particular utility function $u(\cdot)$ that exhibits constant relative risk aversion. Specifically, the lemma establishes the price at which the investors first begin to buy less than the maximum that they can afford.

Lemma S2 (Investor demand less than they can afford iff $\pi > \widetilde{\pi}_{t+1}$)**.** *Suppose that the investors have CRRA utility with parameter λ and that the belief follows a Beta distribution with parameters α_t, β_t. Then under Assumptions S1–S4, we know that $\pi \le \widetilde{\pi}_{t+1}$ implies $D_i(\pi) = y_t/[\pi(1-\mu)]$, and $\widetilde{\pi}_{t+1} < \pi \le \mathbb{E}\, V_{t+1}$ implies $-z_t \le D_i(\pi) < y_t/[\pi(1-\mu)]$.*

Proof. We will show that the first derivative with respect to d of the objective function $g(d; z_t, y_t, c_t, \pi)$ of the maximization in $D_i(\pi)$ evaluated at the upper constraint $d = y_t/[\pi(1-\mu)]$ is negative for $\pi < \widetilde{\pi}_{t+1}$ and positive for $\pi > \widetilde{\pi}_{t+1}$. By Lemma S1, we know that $\partial^2 g(d; z_t, y_t, c_t, \pi)/\partial d^2 < 0$ for all $-z_t \le d \le y_t/[\pi(1-\mu)]$. Because of this negative second derivative, we know that

- $\partial g/\partial d \le 0$ at $d = y_t/[\pi(1-\mu)]$ implies $D_i(\pi) = y_t/[\pi(1-\mu)]$, and that

- $\partial g/\partial d > 0$ at $d = y_t/[\pi(1-\mu)]$ implies $D_i(\pi) < y_t/[\pi(1-\mu)]$,

which proves the claim.

Using $u'(w) = w^{-\lambda}$ in Eq. (S2a) and evaluating at $d = y_t/[\pi(1-\mu)]$ gives

$$\begin{aligned}
\frac{\partial}{\partial d} g(d; z_t, y_t, c_t, \pi)\bigg|_{d=y_t/[\pi(1-\mu)]} &= \int_0^1 u'\left[v\left(z_t + \frac{y_t}{\pi(1-\mu)}\right)\right](v-\pi) f_{t+1}(v)dv \\
&= \left[z_t + \frac{y_t}{\pi(1-\mu)}\right]^{-\lambda} \int_0^1 \left(v^{1-\lambda} - \pi v^{-\lambda}\right) f_{t+1}(v)dv \\
&= \left[z_t + \frac{y_t}{\pi(1-\mu)}\right]^{-\lambda} \left(\mathbb{E}\left[(V_{t+1})^{1-\lambda}\right] - \pi\mathbb{E}\left[(V_{t+1})^{-\lambda}\right]\right),
\end{aligned}$$

which is positive if and only if

$$\pi > \widetilde{\pi}_{t+1} \equiv \frac{\mathbb{E}\left[(V_{t+1})^{1-\lambda}\right]}{\mathbb{E}\left[(V_{t+1})^{-\lambda}\right]} = \frac{\alpha_{t+1} - \lambda}{\alpha_{t+1} + \beta_{t+1} - \lambda}$$

because $z_t + y_t/[\pi(1-\mu)] > 0$ by Assumption S4. This equivalence proves the claim. □

Appendix S2 Derivation of the bank demand function

Here we derive the bank demand function for positive shocks [Eq. (7a)] and for negative shocks [Eq. (7b)] from the original definition [Eq. (4)]. By rearranging the insolvency constraint, Eq. (5), we find that the three constraints (3) are equivalent to

$$-x_t \le d \le \min\left\{\frac{v_{t+1}x_t + r_t - y_t}{\pi - v_{t+1}}, \frac{r_t}{\mu\pi}\right\} \qquad \text{if } \pi \ge v_{t+1}; \tag{S6a}$$

$$\max\left\{-x_t, \frac{v_{t+1}x_t + r_t - y_t}{\pi - v_{t+1}}\right\} \le d \le \frac{r_t}{\mu\pi} \qquad \text{if } \pi < v_{t+1}. \tag{S6b}$$

To evaluate the arg max in Eq. (4), note that the marginal change in the banks' expected equity due to a infinitesimal increase in demand d is

$$\frac{\partial}{\partial_d}\mathbb{E}\, e_{t+1} = \frac{\partial}{\partial_d}\mathbb{E}\left[V_{t+1}(x_t + d) + r_t - (y_t + \pi d)\right] = \mathbb{E}\, V_{t+1} - \pi.$$

Thus, the expected equity $\mathbb{E}\, e_{t+1}$ is linear in d with slope $\mathbb{E}\, V_{t+1} - \pi$, subject to the constraint (S6).

The sign of $\mathbb{E}\, V_{t+1} - \pi$ therefore determines whether the bank demand is the lower or upper constraint in (S6), and the sign of $\pi - v_{t+1}$ determines whether to use constraint (S6a) or (S6b). Note that if $\mathbb{E}\, V_{t+1} < \pi < v_{t+1}$, then the banks' demand is the lower constraint in (S6b), which simplifies to $-x_t$ under the assumption that $\pi x_t + r_t - y_t \geq 0$. In summary, the banks' demand function can be written more explicitly as $D_b(\pi) = -x_t$ if $\pi x_t + r_t - y_t < 0$ or if $\pi > \mathbb{E}\, V_{t+1}$, and otherwise

$$D_b(\pi) = \begin{cases} \dfrac{r_t}{\mu\pi} & \text{if } \pi < v_{t+1} \\ \min\left\{ \dfrac{v_{t+1}x_t + r_t - y_t}{\pi - v_{t+1}}, \dfrac{r_t}{\mu\pi} \right\} & \text{else} \end{cases}. \tag{S7}$$

Finally, considering whether the banks comply with their insolvency constraint (5) immediately after the shock leads to Eq. (7).

Appendix S3 Effect of a capital requirement on the region of beliefs giving rise to three equilibria

Here we explain why, after implementing a capital requirement, the region of beliefs giving rise to three equilibria is reduced from above (and not from below), as illustrated in Fig. 10. Recall that the banks are insolvent if and only if the bond price $\pi \leq (y_t - r_t)/x_t$. Also recall [from the banks' demand in the event of a negative shock, illustrated in Fig. 2(B)] that below the price $(y_t - r_t)/x_t$ the banks are forced to sell all their bonds; by contrast, above the price $(y_t - r_t)/x_t$ and below $\mathbb{E}\, V_{t+1}$, the banks' demand increases with the price. That is, the kink in the bank demand occurs at the price $(y_t - r_t)/x_t$.

Implementing a capital requirement does not affect the location of this kink because the capital constraint $C_{\text{cap. req.}}(\pi)$ [defined in Eq. (10)] satisfies $C_{\text{cap. req.}}[(y_t - r_t)/x_t] = -x_t$. If deposits y_t exceed reserves r_t (as typically occurs in practice), then $C_{\text{cap. req.}}(\pi)$ is increasing in the price π for $\pi \geq (y_t - r_t)/x_t$, so a kink still occurs at the price $(y_t - r_t)/x_t$. On the other hand, if reserves r_t exceed deposits y_t (which rarely occurs in practice), then $C_{\text{cap. req.}}(\pi) > 0$ for all prices π, so the capital requirement does not bind for any price because the bank's demand is negative for a negative shock, and so the bank demand still has a kink at the price $(y_t - r_t)/x_t$. In summary, the position of the first saddle-node bifurcation (at which two new equilibrium prices appear) is $(y_t - r_t)/x_t$ for any minimum capital-to-assets ratio $\gamma_{t+1}^{\min}$; that is, the lower boundary of the region of three equilibria [such as in Fig. 4(E) and in Fig. 10]

is independent of the capital requirement.

Although a capital requirement does not move the location of the kink in the bank demand, a capital requirement can bind (and hence reduce the banks' demand) for prices just above that price where the kink occurs, $(y_t - r_t)/x_t$, as illustrated in Fig. 9(A). Consequently, the left-hand side of the "hump" in the total demand function [depicted in Fig. 4(A)–(C)] is truncated; for an illustration, see Fig. 9(A). Thus, a less severe negative shock causes the two larger equilibrium prices to disappear, as illustrated in Fig. 9(B). In summary, the reduction in the banks' demand just above the price $(y_t - r_t)/x_t$ explains why the region of three equilibria is truncated from above in Fig. 10 and hence why the capital requirement can force a decline in the bond price and bank insolvency.

References

1. Boyd SP, Vandenberghe L (2004) Convex Optimization. Cambridge, UK: Cambridge University Press.
